# Plasma and Serum Alpha-Synuclein as a Biomarker of Diagnosis in Patients With Parkinson's Disease

**DOI:** 10.3389/fneur.2019.01388

**Published:** 2020-01-21

**Authors:** Chun-Wei Chang, Shieh-Yueh Yang, Che-Chuan Yang, Chia-Wen Chang, Yih-Ru Wu

**Affiliations:** ^1^Department of Neurology, Linkou Chang Gung Memorial Hospital, Taoyuan City, Taiwan; ^2^MagQu Co. Ltd., New Taipei City, Taiwan; ^3^Chang Gung University College of Medicine, Taoyuan City, Taiwan

**Keywords:** Parkinson disease, α-synuclein, biomarker, modified Hoehn and Yahr scale, immunomagnetic reduction

## Abstract

**Background:** Parkinson's disease (PD) is the second most common neurodegenerative disease, and α-synuclein plays a critical role in the pathogenesis of PD. Studies have revealed controversial results regarding the correlation between motor severity and α-synuclein levels in peripheral blood from patients with PD.

**Objective:** We examined α-synuclein levels in plasma or serum in patients with PD and investigated the relationship between plasma or serum α-synuclein level and motor symptom severity.

**Methods:** We recruited 88 participants (48 patients with PD and 40 healthy controls). Clinical information was collected, and venous blood was drawn from each participant to be processed to obtain plasma or serum. The plasma or serum α-synuclein level was detected using monoclonal antibodies with magnetic nanoparticles, and was measured through immunomagnetic reduction. Plasma or serum α-synuclein levels were quantitatively detected.

**Results:** In patients with PD, the means of plasma and serum α-synuclein level were 3.60 ± 2.53 and 0.03 ± 0.04 pg/mL, respectively. The areas under the receiver operating characteristic curve of plasma and serum α-synuclein for distinguishing patients with PD from healthy controls were 0.992 and 0.917, respectively. The serum α-synuclein level also showed a significant correlation with patients in H-Y stages 1–3 (*r* = 0.40, *p* = 0.025), implying that the serum α-synuclein level may be a potential marker of motor symptom severity in patients with early PD.

**Conclusions:** Our data suggest that the α-synuclein level in serum or plasma can differentiate between healthy controls and patients with PD. Serum α-synuclein levels moderately correlate with motor severity in patients with early PD.

## Introduction

Parkinson's disease (PD) is the second most common neurodegenerative disease, affecting more than 1% of the global population over the age of 65 years (1). The severity of motor symptoms in patients with PD is commonly evaluated with unified Parkinson's disease rating scale (2) or modified Hoehn and Yahr scale (modified H-Y scale) (3). Because those scales are subjective, there are many objective biomarkers to be developed to diagnose PD or predict disease progression currently. Depending on pathogenesis, many published studies focused on α-synuclein, which is a major constituent of Lewy bodies (4). The majority of researches on levels of different subtype α-synuclein including total (5–7), oligomeric (8, 9), and phosphorylated (9, 10) form in body fluids have been conducted for the cerebrospinal fluid (CSF) (8–14); however, only a few studies have investigated α-synuclein levels in peripheral blood (5–7, 15, 16), and two studies have been done in serum (16, 17). The results of those body fluids between patients with PD and normal control has been controversial (18, 19); additionally, the correlation between the severity of motor symptoms and the levels of α-synuclein in CSF or peripheral blood is still under investigation (7, 9). Therefore, we investigated whether the plasma or serum α-synuclein levels of patients with PD are correlated with motor symptom severity by using a newly developed commercial antibody.

## Materials and Methods

### Ethics Approval and Consent to Participate

This study was approved by the Institutional Review Board of Chang Gung Memorial Hospital (CGMH) in Taiwan (IRB No. 104-7443B) and all examinations were performed after obtaining written informed consents.

### Patient Recruitment

The appropriate number was predicted by G-power, which was a software developed by Universitat Dusseldorf (20). Under preset alpha error (0.05), statistical power (0.9), and medium effect size (0.5), the predicted sample number was 86. On the other hand, Online web-tool, easyROC (http://www.biosoft.hacettepe.edu.tr/easyROC/), was used to predict adequate sample size in ROC curve. Based on alpha error (0.05), statistical power (0.9), area under the ROC curve (0.75), and allocation ratio (1), the predicted minimal sample size (control and case) was 20 and 20, respectively. Thus, we recruited 88 participants, including 48 patients with PD (hereinafter referred to as PDs) and 40 healthy controls (hereinafter referred to as HCs). PD was diagnosed by an experienced neurologist according to the Movement Disorder Society Clinical Diagnostic Criteria for PD (21). We collected clinical information including initial presentation, sex, age, disease duration, cognitive function, and modified H-Y scale scores. According to the modified H-Y scale, patients scoring between 1 and 3 were classified as having early PD, whereas those scoring between 4 and 5 were classified as having advanced PD.

### Plasma and Serum Samples

Venous blood (10 mL) was drawn and blood samples were processed to obtain plasma or serum from each participant within 1 h of collection. Plasma was prepared after collection of the whole blood in Ethylenediaminetetraacetic acid-treated tube, while serum was prepared by leaving blood samples undisturbed at room temperature for 15–30 min. Those processed samples were treated by centrifugation for 15 min at 1,500 g in refrigerated condition, and the resulting supernatant was designated plasma or serum. Following centrifugation, the serum or plasma was immediately transferred into a clean and low residue polypropylene tube using a pipette with low-residue tip. Plasma and serum were stored at −80°C for <3 months before examination. Samples which are hemolyzed, icteric or lipemic were not used.

### Detection and Measurement of Plasma and Serum α-Synuclein in Human Samples

The level of α-synuclein in peripheral blood was examined using the immunomagnetic reduction (IMR) assay. The reagent (MF-ASC-0060, MagQu, Taiwan) used in the assay contained magnetic Fe3O4 nanoparticles (MF-DEX-0060, MagQu, Taiwan) biofunctionalized with monoclonal antibodies which recognizes amino acid residues 121–125 of human α-synuclein (SC-12767, Santa Cruz Biotech, Texas, USA), which was used in a previously study for total α-synuclein measurement (22). The antibody-functionalized magnetic nanoparticles were well-dispersed in phosphate-buffered saline (pH of 7.2). Then, 80 μL of the reagent was mixed with 40 μL of plasma or serum for α-synuclein level measurement by using an alternative current magnetosusceptometer (XacProS, MagQu, Taiwan). The alternative-current magnetic susceptibility of the mixture approximates the association between magnetic nanoparticles and α-synuclein molecules in the plasma or serum. Based on the reduction in the alternative-current magnetic signal of the mixture that was recorded using the analyzer, the α-synuclein level in the plasma or serum could be quantified. Detailed methodologies to immobilize antibodies onto magnetic Fe3O4 nanoparticles, to measure the magnetic concentration of the immunocomplex and to establish a standard curve using liquid form of recombinant human α-synuclein protein (ab51189, Abcam, UK) spiked in phosphate buffered saline between α-synuclein level with and reduction in the alternative-current magnetic signal have been published previously (23). The measurement of the α-synuclein level in plasma or serum was duplicated to improve accuracy.

### Statistical Methods and Data Analysis

Numerical variables were expressed as the mean ± standard deviation. Because of small sample size, the Mann-Whitney U test was used for the comparisons of disease activity between HCs and PDs. A receiver operating characteristic (ROC) curve was applied for distinguishing between the PDs and HCs via the levels of serum or plasma α-synuclein if difference between two groups existed. Additionally, correlation between serum and plasma α-synuclein level, and the relationship between the levels of serum or plasma α-synuclein and disease activity were analyzed using linear regression, and correlation coefficient (*r*) was presented. We performed all analyses using SPSS software, version 24 (IBM, Armonk, NY, USA). A *P*-value of < 0.05 was considered significant.

## Results

Among the recruited patients, the ratio of the men in the HCs (21/40) was similar to that in the PDs (24/48) (*P* = 0.83). The average age of the HCs and PDs was 64.7 and 67.2 years, respectively (*P* = 0.17; Table 1). PDs had mild cognitive impairment (minimal mental status examination: 23.9 ± 5.8), and various degrees of constipation. Levodopa equivalent dose was 869.3 ± 501.2 among PDs.

**Table 1:** Clinical characteristics of the patients with PD and healthy controls.

	**Control (*n* = 40)**	**PD (*n* = 48)**	***P*-value**
Age (years)	64.7 ± 6.8	67.2 ± 9.8	0.17
Gender (male, %)	52.5	50.0	0.83
Duration(years)	N.A.	9.1 ± 6.5	N.A
MMSE	N.A.	23.9 ± 5.8	N.A
Hoehn-and-Yahr stage	N.A.	2.8 ± 1.4	N.A

*Numbers are expressed as mean ± standard deviation. P-values were determined by a parametric t-test and non-parametric chi-square and fisher exact tests*.

*MMSE, Mini-Mental Status Examination; N.A., not available; PD, Parkinson's disease*.

**Figure 1:**
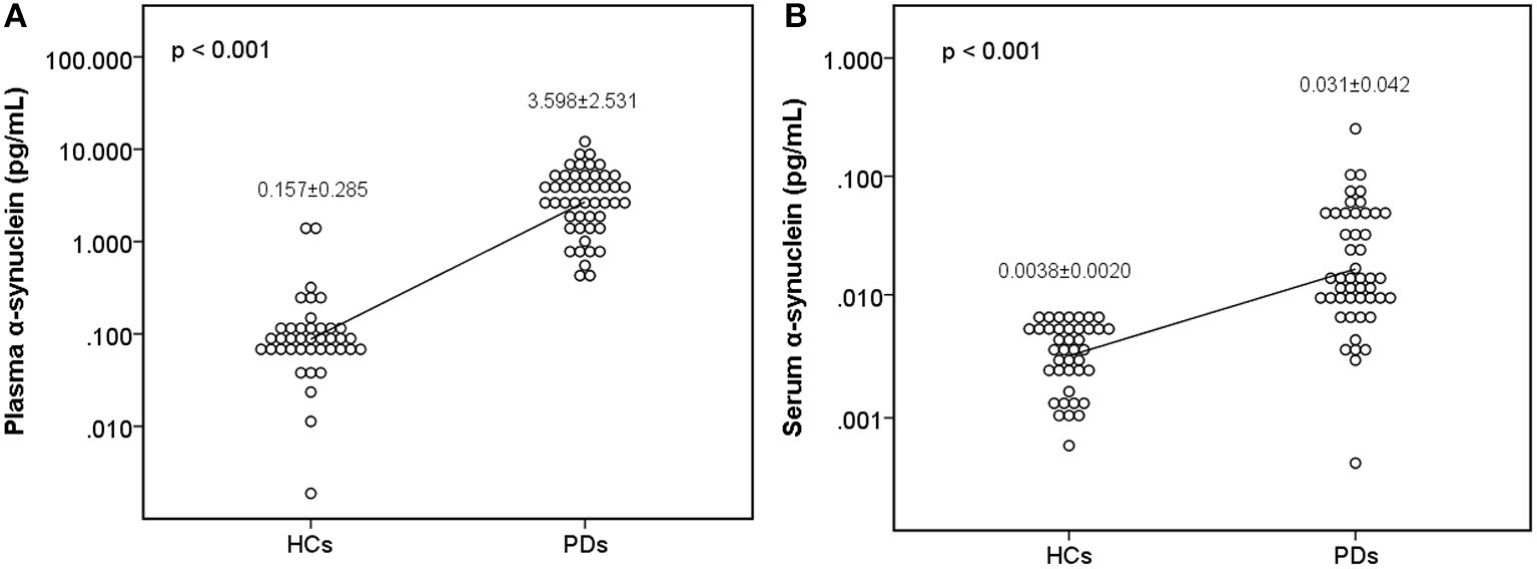
Scatter diagram of plasma α-synuclein levels and serum α-synuclein levels on a logarithmic scale between the healthy control group and the Parkinson's disease group. Significant differences in α-synuclein levels were detected between the two groups in both plasma samples **(A)** and serum samples **(B)**. PD, Parkinson's disease.

**Figure 2:**
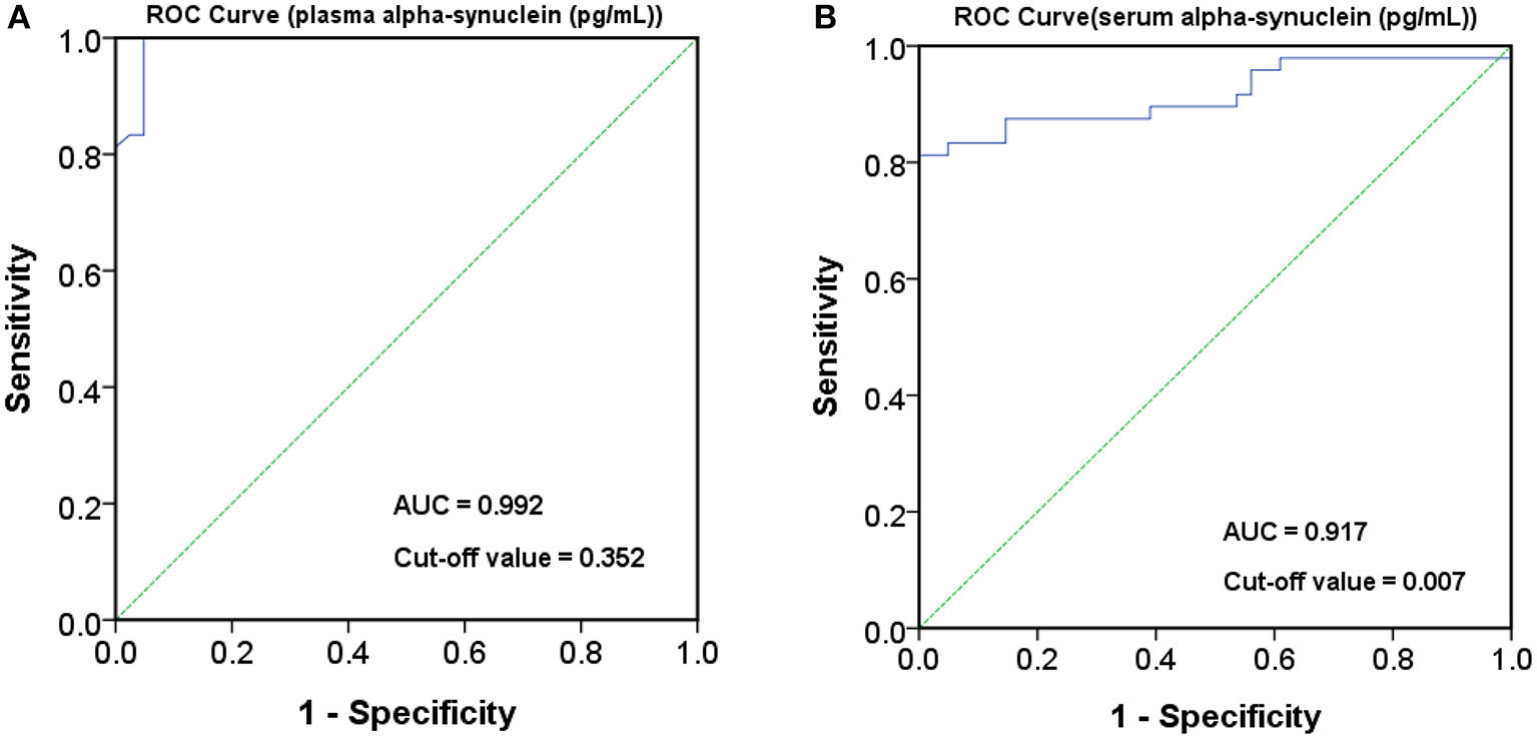
Receiver operating characteristic (ROC) curve for plasma and serum α-synuclein levels to detect Parkinson's disease (PD). ROC curves of plasma **(A)** and serum **(B)** α-synuclein levels for distinguishing PD patients from healthy controls (HCs). AUC, area under the ROC curve.

**Figure 3:**
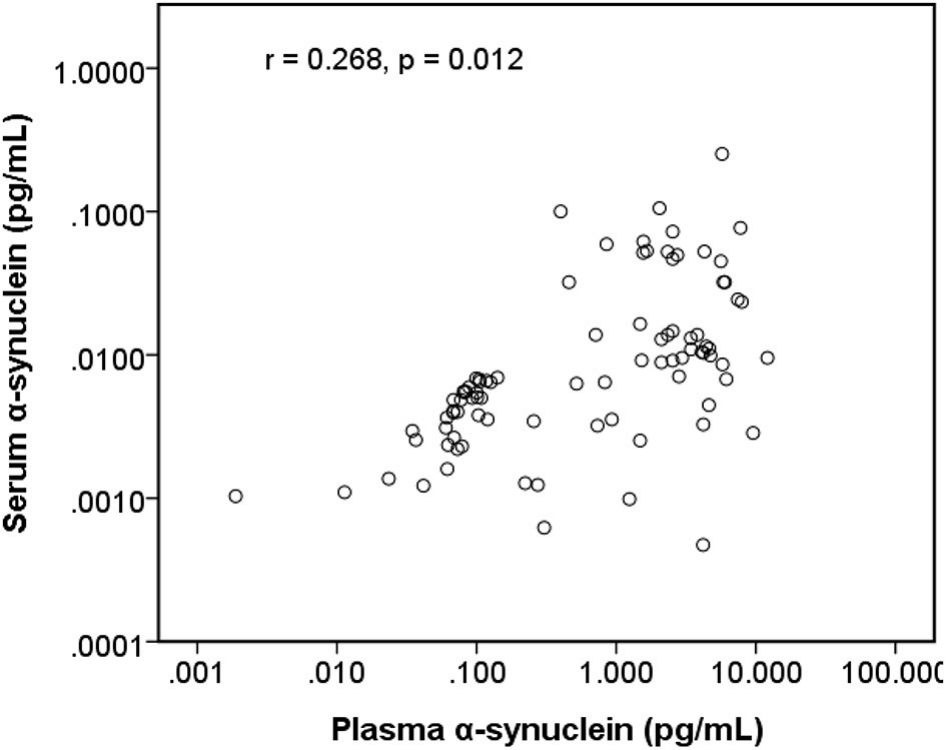
Scatter diagram between plasma α-synuclein levels and serum α-synuclein levels on a logarithmic scale.

The level of plasma α-synuclein in HCs and PDs were 0.157 ± 0.285 pg/mL (coefficient of variance (CV): 11.4%) and 3.598 ± 2.531 pg/mL (CV: 13.7%), respectively (Figure 1A); in contrast, the level of serum α-synuclein in HCs and PDs were 0.0038 ± 0.0020 (CV: 10.9%) and 0.031 ± 0.042 (CV: 13.1%), respectively (Figure 1B). Compared with the HCs, both plasma and serum α-synuclein levels were significantly higher in the PDs (*P* < 0.001 and *P* < 0.001, respectively). The areas under the ROC curve (AUCs) of plasma (Figure 2A) and serum (Figure 2B) α-synuclein levels to distinguish PDs from HCs were 0.992 (cutoff value = 0.352 pg/mL) and 0.917 (cutoff value = 0.007 pg/mL), respectively. A weak correlation was observed between plasma and serum α-synuclein levels, and the correlation coefficient of the linear regression was 0.268 (*P* = 0.012; Figure 3).

**Figure 4:**
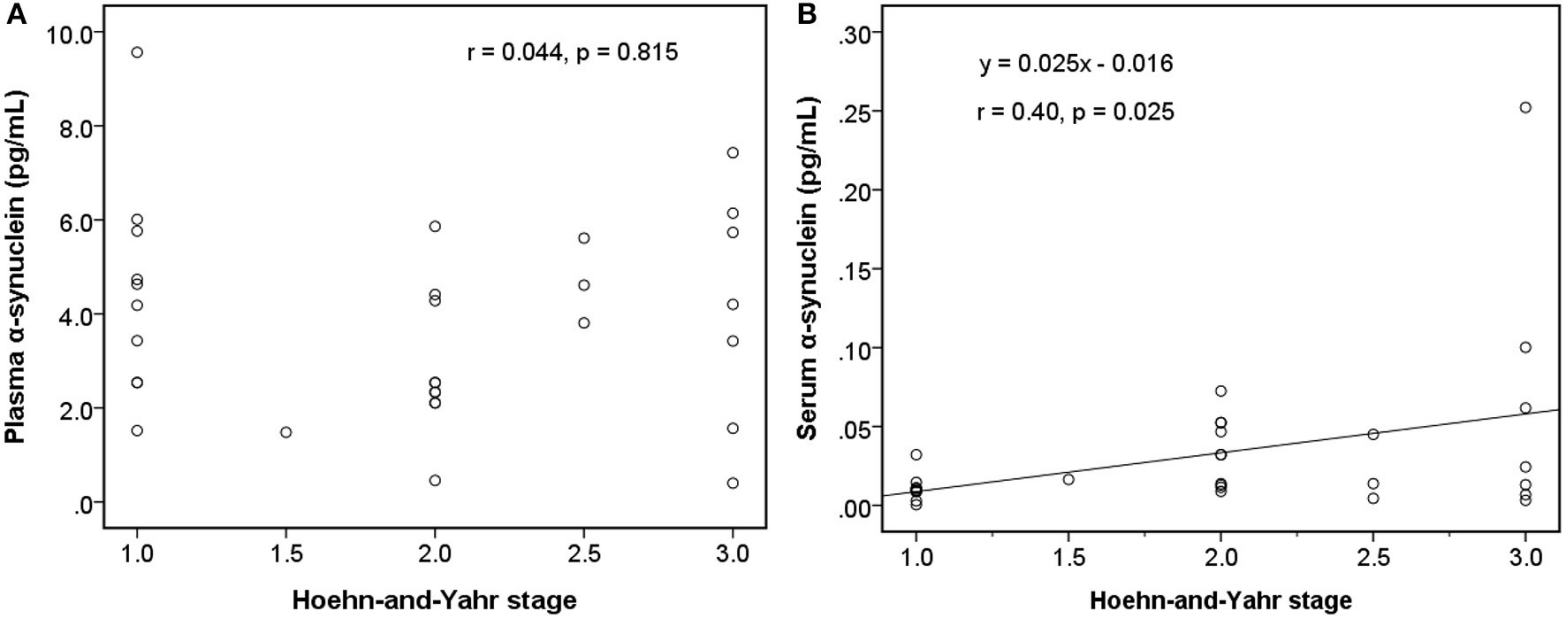
Relationship between α-synuclein levels in plasma or serum samples and clinical severities [modified Hoehn and Yahr stage (H-Y stage)] among patients with early Parkinson's disease (modified H-Y stage from 1 to 3). **(A)** No correlation was detected between α-synuclein levels in plasma samples and modified H-Y stage. **(B)** α-synuclein levels in serum samples and modified H-Y stage were correlated.

**Figure 5:**
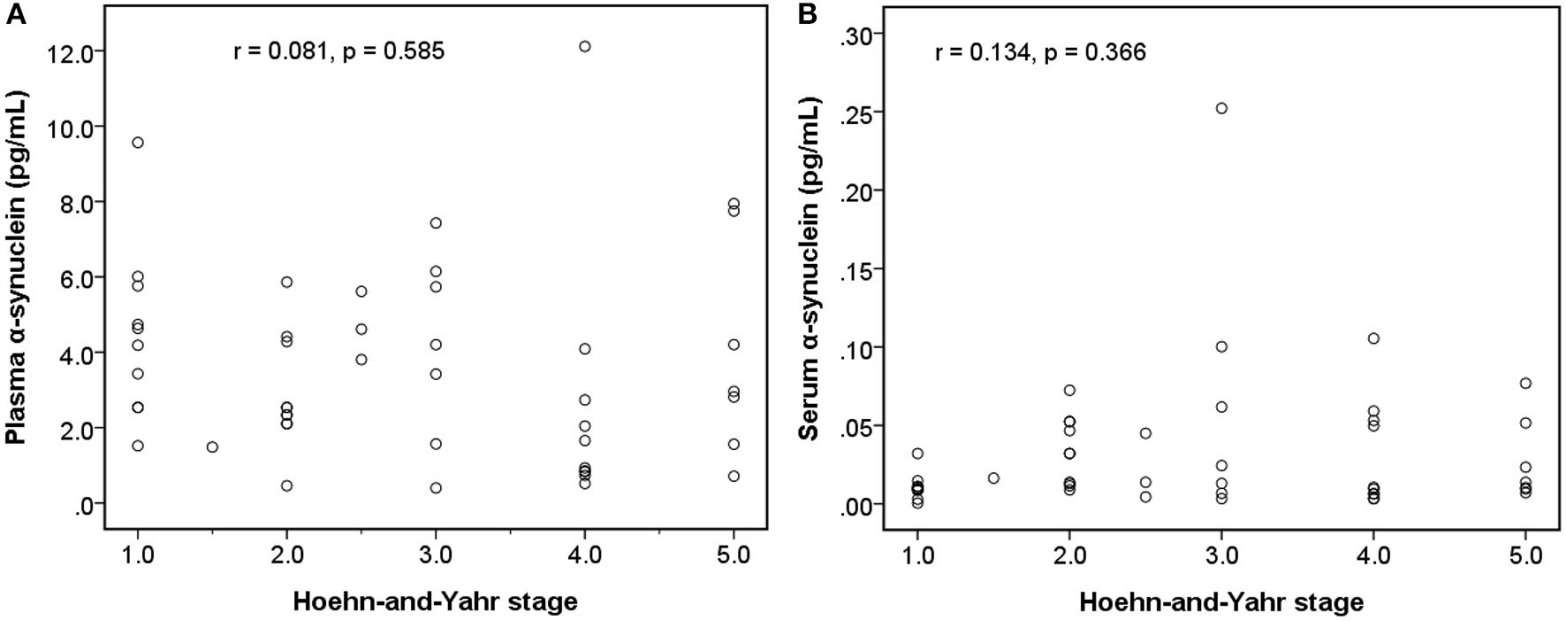
Relationship between α-synuclein levels in plasma or serum samples and clinical severities [modified Hoehn and Yahr stage (H-Y stage)] among patients with Parkinson's disease. **(A)** No correlation was detected between α-synuclein levels in plasma samples and modified H-Y stage. **(B)** No correlation was detected between α-synuclein levels in serum samples and modified H-Y stage.

Among early PDs (modified H-Y stage = 1–3; Figure 4), the level of serum α-synuclein was correlated with modified H-Y stage (*r* = 0.402, *P* = 0.025), whereas plasma α-synuclein was not (*r* = 0.044, *P* = 0.815). Neither plasma (*r* = 0.081, *P* = 0.585) nor serum α-synuclein (*r* = 0.134, *P* = 0.366) correlated with modified H-Y stage in all PDs (Figure 5).

## Discussion

Currently, no reliable biofluid biomarker for distinguishing PDs from HCs has been found. In our study, we demonstrated that not only plasma but also serum α-synuclein levels were higher in the patients with PD than in the HCs through IMR. For the first time, we demonstrated a positive correlation between the serum levels of α-synuclein and the degree of motor symptoms among the patients with early stages of PD. Our observations indicate the potential of serum α-synuclein to be used as an objective biomarker for PD for accurate diagnosis or disease progression monitoring.

α-synuclein, a principal constituent of Lewy bodies, plays a crucial role in the pathogenesis of PD. Because α-synuclein is widely expressed by central and peripheral tissue (24, 25), many studies have targeted different forms of α-synuclein in different samples, such as CSF or blood. Total, oligomeric or phosphorylated α-synuclein levels in CSF could distinguish PDs from HCs (26), and only oligomeric α-synuclein levels in CSF correlated negatively with the severity of motor symptoms (9). By contrast, significant increases in plasma α-synuclein levels have been found in PD patients in previously published studies (5–7, 15, 19). For example, Lee et al. used a commercially available enzyme-linked immunosorbent assay (ELISA) kit to measure the plasma α-synuclein level in subjects with PD. According to the manufacturer's protocol (RPN 5902, Amersham Biosciences, UK), the monoclonal antibody was specific for human synuclein peptide 117–131. The level of α-synuclein was 79.9 ± 4.0 pg/ml in patients with PD (5). Ding et al. used a Chinese sourced commercial ELISA showing higher plasma level of α-synuclein (319.56 ± 64.22 vs. 274.31 ± 70.71, *p* = 0.004) than controls (6). Foulds et al. reported vastly different results using same monoclonal antibody in individual subjects (15), which might be due to their recombinant standard was highly impure. According to the published papers, the commonly used technologies for assaying plasma α-synuclein included bead-based multi-analyte profiling technology (Luminex), sandwiched ELISA or IMR (19). However, these reports showed highly inconsistent results in levels of plasma α-synuclein. Mata et al. and Shi et al. used Luminex assays to evaluate plasma α-synuclein in PD; and the levels were 46.9 ± 32.6 and 36.8 ± 23.9 ng/mL, respectively (27, 28). The antibodies used in both studies were biotinylated anti-human α-synuclein antibody (R&D systems, Minneapolis, MN, USA). It is unclear, however, whether these α-synuclein species were oligomers or monomers conjugated with other macromolecules.

Moreover, Wang et al. used the same kit to measure plasma α-synuclein and found sub-ng/ml levels in PD and HC (29). The principal origin of α-synuclein are red blood cells (RBCs) (>99% of its blood levels), with the residue in plasma. Hemolysis and platelet contamination confound the results. The inconsistence in levels of plasma α-synuclein among groups using the same assay kits might be possibly caused by plasma preparation and storage period of plasma samples. Groups using ELISA technologies reported several tens of pg/ml, or thousands of ng/ml for plasma α-synuclein in PD and HC (5, 15). The levels of plasma α-synuclein using ELISA are different from that using Luminex. This difference could be due to antibodies, signal sensing technologies and sample preparation. Therefore, we could also expect that high heterogeneity across studies could be attributed to the co-existence of several components such as assays, disease duration, disease staging, and study setting.

Thus, the levels of α-synuclein in plasma measured by IMR could be different from those using Luminex or ELISA. In this study, the levels of α-synuclein in plasma are pg/ml, which is consistence with previous work using IMR (7). Moreover, the discrimination between PD and HC using the levels of plasma α-synuclein is clear in this and previous works (5, 23). These results reveal the high reliability of detected levels of plasma α-synuclein using IMR, although the levels using IMR are much lower than that using Luminex or ELISA.

The potential mechanism underlying increased plasma and serum levels of α-synuclein in patients is still unclear. α-synuclein is a product of *SCNA* gene in neurons, erythrocytes, lymphocytes, and enteroendocrine cells (25). The protein is released from neurons through exocytosis and membrane leakage such as apoptosis, necrosis, or other damage (30). According to gut-brain axis of PD, misfolded or toxic α-synuclein is originated from the peripheral enteric plexus. Therefore, the increase in plasma and serum α-synuclein levels may be attributed to peripheral origin including enteric plexus or erythrocyte in early stage PD (31, 32). Along with disease progression, abnormal erythrocyte-derived and peripheral neuron-derived α-synuclein migrates to the brain, and then deposits (33). The α-synuclein can be removed from the brain through exocytosis with exosomes, and the exosomes containing α-synuclein and specific surface markers derived from the brain can be found in peripheral blood (34). Excess α-synuclein in the brain may trigger efflux of the protein from the CSF to blood; thus, the α-synuclein level increased in plasma and serum but decreased in the CSF (35, 36). The α-synuclein level is higher in blood than in the CSF; therefore, its transport from the CSF to blood may be energy dependent for concentration gradient (37). Because of the limited number of pump and energy for efflux of excess α-synuclein, serum, and plasma α-synuclein level may become steady in the late stage of PD and excess α-synuclein deposited in brain parenchyma. This possible can explain why serum and plasma α-synuclein levels could distinguish patients with PD from HCs, whereas they could not correlate with motor symptom severity in the late stages of PD.

Another critical finding in our study was that serum α-synuclein levels showed a positive correlation with motor symptom severity in patients in the early stages of PD, whereas plasma α-synuclein level did not. The plasma α-synuclein level was significantly higher than the α-synuclein level in serum. Higher plasma α-synuclein level may be attributed to cell lysis of free erythrocyte and platelets (38), and to more α-synuclein-containing exosomes from free erythrocytes (33), whereas serum might contain fewer exosomes because of erythrocytes that are trapped in the fibrin complex. Furthermore, proteases such as plasmin in platelet activation cleaved free α-synuclein from cell lysis in serum (39) during clot formation; by contrast, materials in exosome may be protected (40). As a result, serum contained less erythrocyte-derived α-synuclein in the free form or in the exosome. The correlation between the levels of plasma and serum α-synuclein became week, and serum α-synuclein, which contained more CNS-derived α-synuclein in exosome, may reflect the α-synuclein burden in the CNS more accurately.

This study has several limitations. First, this is a cross-sectional study, so a longitudinal study is needed to keep track of the plasma or serum α-synuclein levels in one subject to disclose the change of α-synuclein level in plasma or serum over the time during the disease progression. Second, the sample size of the study is still relatively small; therefore, a larger and multi-center study discovering the relationship between α-synuclein in peripheral blood and disease activity is necessary to validate the practicality of using plasma and serum α-synuclein as a reliable biomarker for PD. Third, because the selected antibody in our study only identify very short amino acid sequence 121–125 of α-synuclein, alpha-synuclein with epitope modified by polymerization, methylation, phosphorylation, or other chemical reaction could be not detected in the study. Moreover, measuring sub-picograms of protein may be influenced significantly by measurement bias and cross-reactivity of a selected antibody. Further studies using another commercial antibody through the same assay is necessary to validate this new technique and to reduce the influence of cross-reactivity.

In conclusion, our data suggests that α-synuclein levels in serum or plasma can differentiate between HCs and patients with PD. Serum α-synuclein levels moderately correlated with motor symptom severity in patients with early PD. A larger, multicenter study is necessary to investigate the mechanism underlying α-synuclein aggregation and the relationship between α-synuclein and disease progression.

## Ethics Statement

This study was approved by the Institutional Review Board of Chang Gung Memorial Hospital (CGMH) in Taiwan (IRB No.104-7443B) and all examinations were performed after obtaining written informed consents.

## Author Contributions

Y-RW conceived and designed the study. S-YY, C-CY, and C-WenC conducted the experiments. C-WeiC and Y-RW analyzed the data and wrote the paper. All authors read and approved the final manuscript.

### Conflict of Interest

S-YY is an employee at MagQu Co., Ltd. and holds stock shares of MagQu. C-CY has been an employee at MagQu Co., Ltd., and is resigned now. The remaining authors declare that the research was conducted in the absence of any commercial or financial relationships that could be construed as a potential conflict of interest.
